# Multicentre randomised trial comparing contact force with electrical coupling index in atrial flutter ablation (VERISMART trial)

**DOI:** 10.1371/journal.pone.0212903

**Published:** 2019-04-03

**Authors:** Gordon A. Begg, James O’Neill, Afzal Sohaib, Ailsa McLean, Chris B. Pepper, Lee N. Graham, Andrew J. Hogarth, Stephen P. Page, Richard G. Gillott, Nicola Hill, Jacqueline Walshaw, Richard J. Schilling, Prapa Kanagaratnam, Muzahir H. Tayebjee

**Affiliations:** 1 Department of Cardiology, Leeds Teaching Hospitals NHS Trust, Leeds General Infirmary, Leeds, United Kingdom; 2 Department of Cardiology, Imperial College Healthcare NHS Trust, Hammersmith Hospital, London, United Kingdom; 3 Department of Cardiology, Barts Health NHS Trust, St Bartholomew’s Hospital, London, United Kingdom; University of Messina, ITALY

## Abstract

**Introduction:**

Electrical coupling index (ECI) and contact force (CF) have been developed to aid lesion formation during catheter ablation. ECI measures tissue impedance and capacitance whilst CF measures direct contact. The aim was to determine whether the presence of catheter / tissue interaction information, such as ECI and CF, reduce time to achieve bidirectional cavotricuspid isthmus block during atrial flutter (AFL) ablation.

**Methods:**

Patients with paroxysmal or persistent AFL were randomised to CF visible (range 5-40g), CF not visible, ECI visible (change of 12%) or ECI not visible. Follow-up occurred at 3 and 6 months and included a 7 day ECG recording. The primary endpoint was time to bidirectional cavotricuspid isthmus block.

**Results:**

114 patients were randomised, 16 were excluded. Time to bidirectional block was significantly shorter when ECI was visible (median 30.0 mins (IQR 31) to median 10.5mins (IQR 12) p 0.023) versus ECI not visible. There was a trend towards a shorter time to bidirectional block when CF was visible. Higher force was applied when CF was visible (median 9.03g (IQR 7.4) vs. 11.3g (5.5) p 0.017). There was no difference in the acute recurrence of conduction between groups. The complication rate was 2%, AFL recurrence was 1.1% and at 6 month follow-up, 12% had atrial fibrillation.

**Conclusion:**

The use of tissue contact information during AFL ablation was associated with reduced time taken to achieve bidirectional block when ECI was visible. Contact force data improved contact when visible with a trend towards a reduction in the procedural endpoint.

ClinicalTrials.gov trial identifier: NCT02490033.

## Introduction

Until recently, catheter contact during ablation was determined using surrogates such as lack of catheter motion, electrogram attenuation and electrical impedance drops [1,2]. However, the importance of being able to quantify the catheter-tissue interface has become increasingly clear [3] and there are now technologies available which allow the direct measurement of tissue catheter contact and tissue impedance and capacitance.

The Thermocool Smart Touch Catheter used in conjunction with the CARTO-3 three-dimensional (3D) mapping system (Biosense Webster, Diamond Bar CA, US) has a spring at the tip of the ablation catheter which deforms as pressure is applied; this measures contact force (CF) directly and can prevent an operator from from applying excessive amounts of force. The Ensite Verisense System used in conjunction with the Ensite NAVx Velocity 3D mapping system (St Jude Medical, St Paul MN, US) calculates the electrical coupling index (ECI) which is derived from tissue resistance and reactance [4,5]. The measurement of ECI does not provide direct information on tissue contact, but the catheter is calibrated at the start of the case to determine an individual patient’s ECI range for viable tissue. A low ECI value can either mean poor tissue contact or ablated tissue. Therefore ablation is performed within a pre-specified ECI range implying viable tissue and some catheter contact. In contrast, CF catheters give direct information about the tissue/catheter interface, but do not provide data on tissue viability.

Catheter ablation for atrial flutter (AFL) is well-established and more effective than cardioversion or rate-control therapy [6]. Acute procedural success, defined by bidirectional isthmus block, is over 95% with a longer term recurrence rate of 5–10% [7]. The principle reason behind this is recovery of isthmus conduction [8]. Novel techniques/technologies are constantly being developed. However it is important that these are tested to ensure efficacy and safety as theoretical or in vitro benefit does not necessarily equate to clinical success. As ablation of AFL is well-established and provides consistent results, unlike ablation for atrial fibrillation (AF) for example (as the anatomy of the cavotricuspid isthmus is relatively consistent from patient to patient, and the linear ablation lesion relatively simple), we chose to study this procedure rather than more complex ablations in order to reduce confounding. The study is not designed to assess improvements in the efficacy of ablation with respect to flutter recurrence, moreover to examine the relative effects of the contact technologies on the time taken to create an effective lesion set.

We therefore hypothesised that during flutter ablation, additional data about the catheter/tissue interface would reduce the time taken to achieve bidirectional block compared to using 3D mapping alone. The term *contact technology* will be used as a way of describing both CF and ECI.

## Methods

### Study design

The VERISMART study was an investigator-initiated, multicentre, prospective, randomised trial comparing CF, ECI, and non-contact, 3D-guided ablation where contact technology information was not visible to the operator. We recruited patients with symptomatic persistent or paroxysmal AFL who had been listed for their first attempt at catheter ablation between 7^th^ April 2015 and 5^th^ February 2017. All patients gave written informed consent and the study had ethical approval from the Health Research Authority and the Yorkshire & The Humber—Bradford Leeds Research Ethics Committee (REC number 14/YH/0038). It was registered with clinicaltrials.gov (trial identifier: NCT02490033).

### Eligibility criteria

Patients were included if they were aged 18 years or older and if they had documented evidence of persistent or paroxysmal AFL. Exclusion criteria were an inability or unwillingness to receive oral anticoagulation, a previous ablation for AFL, concomitant AF, current participation in another ablation study or an inability to complete the required study follow-up.

### Randomisation

The treatment groups were CF visible, CF not visible, ECI visible and ECI not visible. Block randomisation with sealed envelopes was used to allocate the treatment groups. Envelopes were opaque and identical and contained a card with the treatment group printed. The envelopes were only opened at the time of participant enrolment and the sequence of randomisation was concealed from both clinical staff and patients to avoid potential selection bias.

### Study protocol

The ablation procedures took place in three high-volume institutions by a total of seven experienced cardiac electrophysiologists familiar with both contact technologies. They were performed with patients in the post-absorptive state under conscious sedation or general anaesthesia. Oral anticoagulation was continued peri-procedure; CARTO-3 or NavX Velocity were used as the 3D mapping systems for Thermocool Smart Touch (Biosense Webster, Diamond Bar CA, US) CF catheters and Ensite Verisense (St Jude Medical, St Paul MN, US) ECI catheters respectively. Venous sheaths were inserted into the right and/or left femoral veins. No steerable sheaths were used. A decapolar catheter was inserted into the coronary sinus. An additional 20-pole halo diagnostic catheter (Biosense Webster, Diamond Bar CA, US) could be placed around the tricuspid valve annulus or a quadripolar catheter into the right ventricle as per operator preference. The creation of three dimensional right atrial geometry and activation mapping was left to the discretion of the operator.

If the patient was randomised to one of the non-visible control arms, the contact information was withheld from the operator. For CF, this was accomplished by removing the CF window. This was not possible for ECI and so an opaque blanking material was physically placed over a section of the operator’s display, covering the ECI scrolling waveform and data, but no other information. The beacon displayed over the catheter tip on the mapping system colour coded for ECI was disabled.

A cavotricuspid-isthmus line was created by either a drag approach, point by point ablation, or both according to operator preference using the CF or ECI catheters. Energy was delivered at each point for 30 seconds with flow limited to 17ml/min and power limited to 40W with a maximum temperature of 48°C. If bidirectional block was not achieved on the first pass, additional ablation targeting gaps were applied. Bi-directional isthmus block was confirmed by measuring conduction interval from a pacing stimulus in the distal coronary sinus to a local electrogram recorded by the ablation catheter placed either side of the isthmus line. This pacing manoeuvre was then reversed, and conduction interval to the coronary sinus while pacing on the tricuspid annulus was measured. Finally, under CS pacing, conduction interval to progressively more lateral sites around the isthmus was confirmed to decrease. Following bidirectional block, a waiting time of 30 minutes was observed to check for isthmus re-conduction. In the CF group, individual ablation lesions were manually created on the mapping system. When a point–by–point technique was used, a lesion was created for each 30 second RF application. When a drag technique was used, a lesion was manually created after every 30 seconds of RF application. The mapping system could then be interrogated retrospectively to record the average CF applied during each 30-second lesion. In each case, the length of the ablation line was measured. The line was then divided into three equal segments–tricuspid annulus, middle, and inferior vena cava–for analysis of forces applied at different anatomical sites.

For the CF group, a CF range of 5–40g was recommended. For safety, operators would be warned if CF exceeded 50g to reduce the risk of cardiac perforation, irrespective of the randomisation arm. In the ECI group, a change in ECI of 12% was aimed for as this has previously been shown to be the optimal value for safe full thickness lesion formation [9]. In the ECI visible group, a 30 second ablation was completed even if a 12% reduction in ECI was achieved within this period of time.

All anti-arrhythmic medications were discontinued following the procedure but anticoagulation was maintained for a minimum of two months. Follow-up occurred at 3 and 6 months and included a 12-lead electrocardiogram (ECG) at each visit together with a 7-day ECG recording at the end of the study to assess for paroxysmal atrial arrhythmias (>30 seconds).

### Outcomes

The primary outcome of the study was time taken to achieve bidirectional block (defined as the time from the onset of energy delivery at the first lesion to the time that consistent (> one minute) bidirectional block was achieved). Secondary outcomes were total radiofrequency (RF) time for the whole procedure, total energy required for ablation (defined as power x ablation time), total procedure time, fluoroscopy time during ablation and total fluoroscopy time.

### Sample size

A sample size of 120 patients was chosen for the study, aiming for 30 participants in each treatment group. This was based upon published guidance on the design of pilot studies and from numbers used in previous studies [10].

### Statistical analysis

Continuous variables were checked for normality using the Kolgorov-Smirnoff test. If they were normally distributed the results were expressed as mean ± standard deviation and compared by Student’s t-test or ANOVA. If they were skewed the results were expressed as median and interquartile range and compared using logarithmic-transformed ANOVA or, if transformation was not possible, the Mann Whitney U Test or Kruskal-Wallis Test. Tukey’s test was used for post-hoc analysis if the ANOVA result was significant. A chi-squared test was used to compare categorical variables. A p value <0.05 was considered statistically significant.

## Results

**Fig 1:**
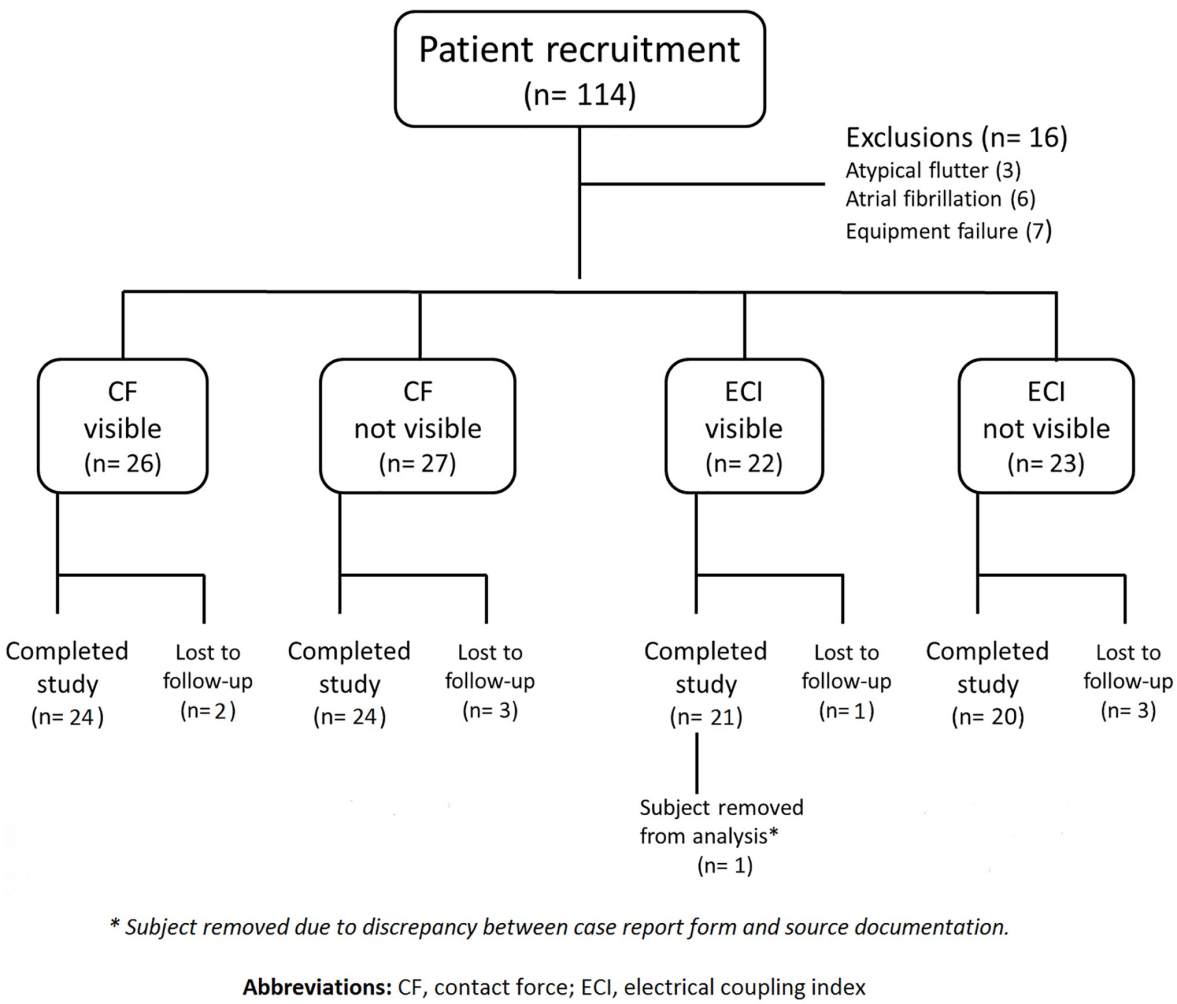
Study recruitment flowchart.

A total of 114 patients were randomised into the trial between April 2015 and February 2017 (Fig 1). Of these, 16 patients were excluded from the study, either due to the finding of AF or atypical (non-isthmus dependent) atrial flutter at the time of the procedure or due to equipment failure. A further 9 patients were lost to follow-up. One patient was excluded from analysis due to a discrepancy between the case report form and source documentation whereby activation mapping had been used during the case but this had not been recorded in the case report form. It was not possible to ascertain whether the time to bidirectional block included activation mapping and so the data was removed from analysis.

There was no difference in the baseline characteristics between the 4 treatment groups (Table 1).

**Table 1:** Baseline characteristics.

	CF not visible (n = 27)	CF visible (n = 26)	ECI not visible (n = 23)	ECI visible (n = 22)	P value
Age	65.3 (16.5)	62.7 (21.2)	63.5 (12.3)	64.0 (17.1)	0.757
Male gender	21 (77.8)	24 (92.3)	20 (87.0)	19 (86.4)	0.503
Body mass index	28.5 (7.0)	29.1 (6.0)	28.9 (8.4)	28.4 (6.4)	0.937
Time since first documented atrial flutter (days)	467 (1069)	271 (374)	287 (371)	294 (312)	0.367
Duration of persistent atrial flutter (months)	7.5 (9.0)	6.0 (5.0)	5.0 (7.0)	5.5 (11.0)	0.473
Persistent atrial flutter	18 (66.7)	19 (73.1)	10 (43.5)	14 (63.6)	0.173
Flutter at start of ablation	16 (59.2)	16 (61.5)	10 (43.5)	9 (40.9)	0.352
CHA_2_DS_2_VASc					
0	5 (18.5)	5 (19.2)	7 (30.4)	8 (36.4)	0.333
1	6 (22.2)	7 (26.9)	6 (26.1)	4 (18.2)	
2	10 (37.0)	3 (11.5)	4 (17.4)	5 (22.7)	
3	1 (3.7)	6 (23.1)	2 (8.7)	3 (13.6)	
4	3 (11.1)	4 (15.4)	4 (17.4)	2 (9.1)	
5	2 (7.4)	0	0	0	
6	0	0	0	0	
7	0	1 (3.8)	0	0	
Ischaemic heart disease	3 (11.1)	8 (30.8)	5 (21.7)	5 (22.7)	0.380
Hypertension	11 (40.7)	12 (46.2)	8 (34.8)	7 (31.8)	0.743
Heart failure	4 (14.8)	6 (23.1)	1 (4.3)	1 (4.5)	0.135
Valvular heart disease	2 (7.4)	0	2 (8.7)	2 (9.1)	0.498
Dilated cardiomyopathy	2 (7.4)	0	2 (8.7)	1 (4.5)	0.508
Hypertrophic cardiomyopathy	1 (3.7)	0	0	0	0.448
Chronic kidney disease	2 (7.4)	1 (3.8)	1 (4.3)	1 (4.5)	0.936
Cerebrovascular disease	0	1 (3.8)	1 (4.3)	0	0.561
Diabetes	5 (18.5)	7 (26.9)	3 (13.0)	1 (4.5)	0.199
Hypothyroidism	1 (3.7)	0	0	1 (4.5)	0.555
Peripheral vascular disease	0	2 (7.7)	0	0	0.130
Amiodarone	3 (11.1)	3 (11.5)	2 (8.7)	2 (9.1)	0.984
Flecainide	0	1 (3.8)	1 (4.3)	0	0.561
Beta blocker	22 (80.5)	19 (73.1)	13 (56.5)	15 (68.1)	0.337
Calcium channel antagonist	2 (7.4)	4 (15.4)	2 (8.7)	2 (9.1)	0.782

Values displayed are medians (interquartile range). Statistical analysis involved Kruskal-Wallis for continuous variables or Chi-squared test for categorical variables

*Abbreviations*: CF, contact force; ECI, electrical coupling index

**Fig 2:**
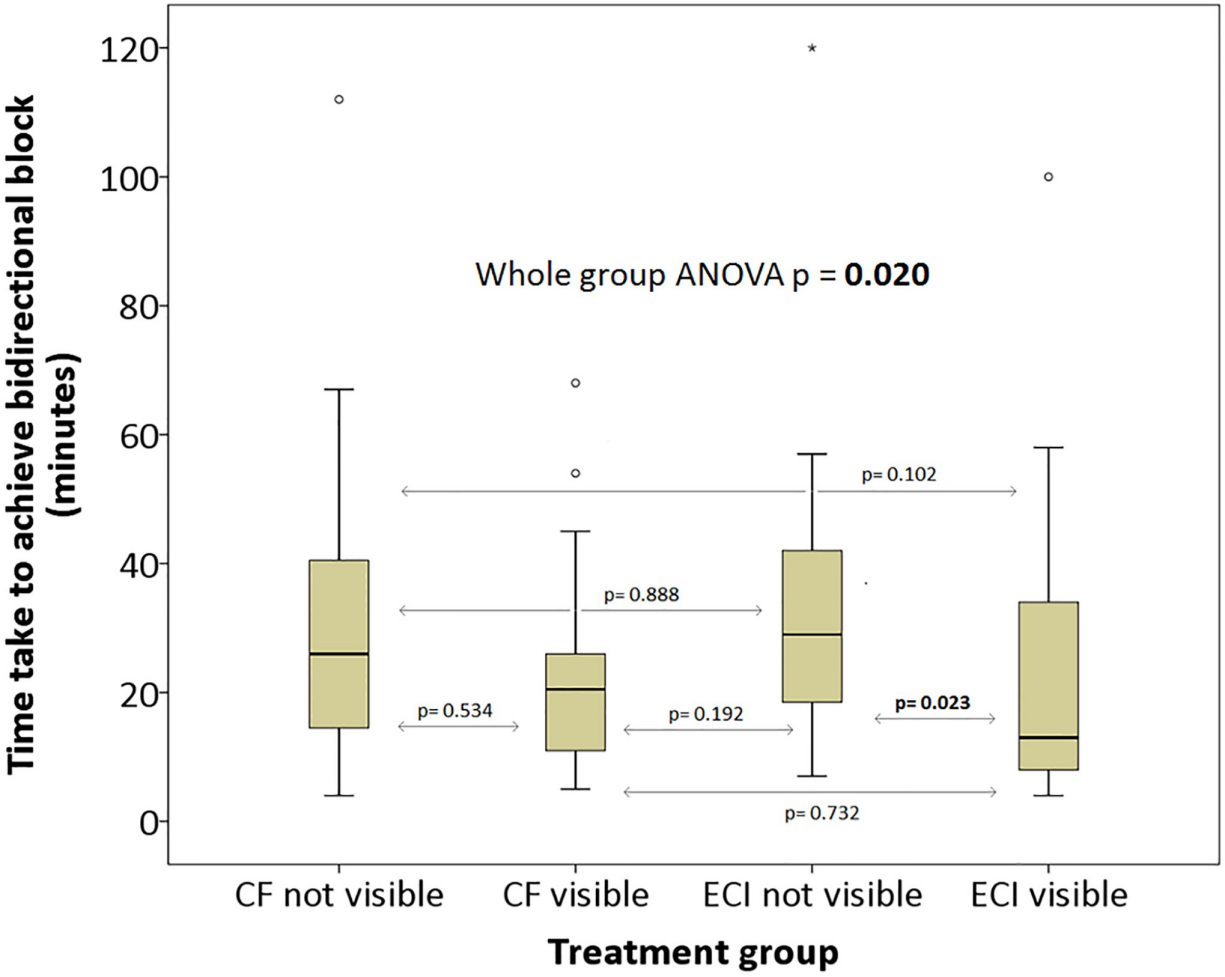
Boxplot of time taken to achieve bidirectional block in different treatment groups (p values represent Tukey’s between-group analyses).

Time to achieve bidirectional block was significantly shorter in the ECI visible group compared to ECI not visible (Fig 2). Although time to bidirectional block was shorter in the CF visible compared to CF not visible, this was not statistically significant (Table 2). There was no difference between the groups where CF and ECI were not visible.

**Table 2:** Primary and secondary outcomes in treatment groups.

	CF not visible	CF visible	ECI not visible	ECI Visible	P value
**Time to achieve bidirectional block (minutes)**	26.0 (25)	20.5 (15)	30.0 (31)	10.5 (12)	**0.020**
	0.534		**0.023**		
**Total RF time (seconds)**	667 (521)	561 (811)	1131 (714)	641 (427)	0.076
	0.999		0.319		
**RF time power product (watt-seconds/1000)**	26.4 (20.8)	22.4 (32.9)	45.2 (27.4)	25.2 (17.1)	0.066
	1.000		0.300		
**Fluoroscopy time during CTI line creation (seconds)**	238 (219)	137 (189)	311 (387)	123 (177)	**0.004**
	0.873		**0.002**		
**Total fluoroscopy time (seconds)**	507 (374)	393 (348)	866 (581)	492 (510)	**0.019**
	0.977		0.389		
**Total procedure time (minutes)**	80.0 (33.5)	73.0 (33.3)	97.0 (23.0)	74.0 (19.0)	0.138
	0.978		0.096		
**30-minute success rate (number of cases)**	22 [81.4]	22 [84.6]	20 [87.0]	18 [81.8]	0.957
	0.990		0.968		
**Length of line (millimetres)**	33.0 (17.0)	33.0 (15.5)	37.0 (6.00)	34.5 (14.7)	0.084
	0.999		0.882		
**Average overall force (grams) (total)**	9.03 (7.43)	11.3 (5.54)			**0.016**
**Average CF force (grams) (IVC)**	7.40 (12.3)	12.5 (7.68)			**0.032**
**Average CF force (grams) (mid)**	9.10 (7.20)	12.4 (6.23)			**0.032**
**Average CF force (grams) (TVA)**	6.50 (4.90)	10.6 (6.55)			**0.003**

Values shown are medians (interquartile range). Statistical analysis involved logarithmic transformed ANOVA and post-hoc Tukey’s Test.

**Abbreviations:** CF, contact force; ECI, electrical coupling index; RF, radiofrequency; CTI, cavotricuspid isthmus; IVC, inferior vena cava; mid, mid-cavotricuspid isthmus; TVA, tricuspid valve annulus

There was no significant difference in total RF energy application time, total energy required for ablation or total procedure time when CF or ECI data was available (it should be noted that this time period included a 30 minute waiting period and further ablation if required; Table 2). Total fluoroscopy time was higher in the ECI treatment group than the CF treatment group (Table 2).

**Fig 3:**
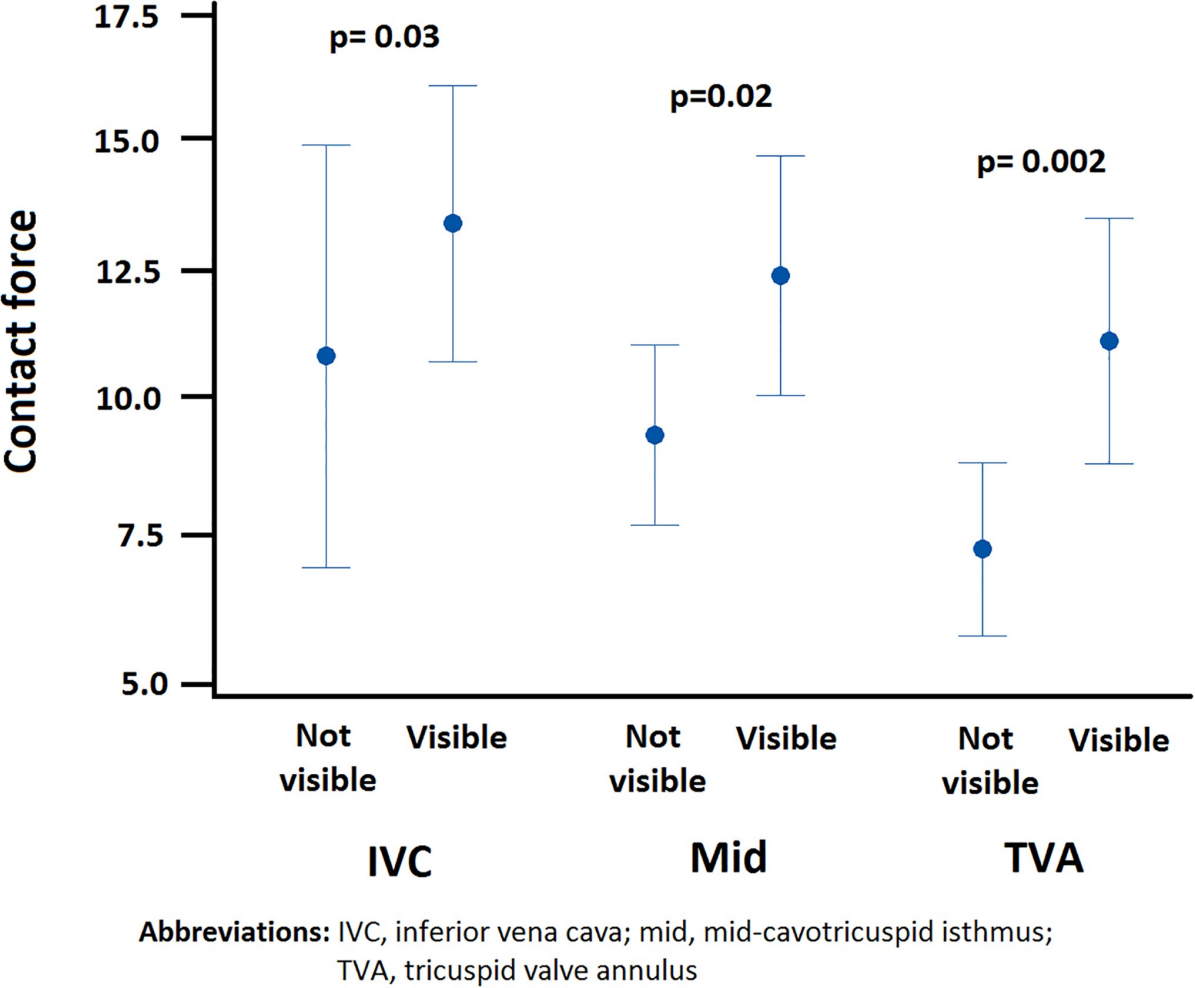
Applied contact force with CF visible and CF not visible.

In the CF group, the median average force that was applied at the level of the inferior vena cava, mid-isthmus and tricuspid valve annulus was significantly higher when CF was visible (Fig 3). Average CF force was lowest at the level of the tricuspid valve annulus.

There was no significant difference in the acute recurrence of conduction between the 4 treatment groups (p = 0.957). The overall complication rate for the study was 2%. A subject in the ECI blinded arm experienced a transient ischaemic attack 24 hours after the ablation procedure whilst a subject in the ECI unblinded arm had ventricular fibrillation (which was treated with a single DC shock) as a consequence of catheter-induced ventricular ectopy.

At the end of 6 month follow-up, AFL recurrence rate was 1.1% and AF occurred in 12% of subjects who completed the study. If those patients with AF who were excluded at the time of the procedure are included into this figure, the total AF occurrence for the study population was 15.8%.

## Discussion

This study is the first head to head comparison of two different technologies that provide information on the interaction between the ablation catheter and cardiac tissue in flutter ablation. The use of contact technology (in this study, ECI) increases catheter/tissue contact, reduces the time to achieve the primary endpoint of bidirectional block when ECI is visible and reduces the need for X-ray.

Contact force and ECI are very different. The Thermocool Smart Touch catheter (Biosense Webster, Diamond Bar, CA, USA) incorporates a spring between the electrode and catheter shaft. A precision spring within the catheter tip allows the measurement of movement and deflection between the shaft and tip electrode [11]. As such, it is a direct measurement of CF but provides no information on tissue viability. The Ensite Verisense (St Jude Medical, St Paul MN, US), does not measure contact force, but provides data on the tissue catheter interaction using a proprietary algorithm based on the resistance and reactance of the underlying tissue [4,12]. The ECI therefore reflects a composite of contact (but not quantitatively as in the case of CF) and tissue viability. Therefore, the ECI may be low in an area of a full thickness scar with good contact, or in an area of viable myocardium with poor contact. Ablation would generally not be indicated in either of these situations, and the catheter should be moved to a different area.

Contact sensing technology has the potential to assist in the delivery of effective ablation lesions. In aiming to demonstrate this concept, atrial flutter ablation was chosen because the cavotricuspid isthmus is less anatomically variable between patients than other regions of the heart (the left atrium or ventricle, for example), and the ablation lesion is based on anatomical landmarks. This leads to a more consistent lesion from patient to patient than is the case with other common ablations. Furthermore, the cavotricuspid isthmus lesion is substantial enough to give a meaningful quantity of data for subsequent analysis, contrasting with the smaller, focussed lesions required for pathway or nodal ablation. Therefore, confounding anatomical and functional factors were minimised. This is evidenced by the fact that the length of the ablation line was not significantly different in the four groups.

In patients where CF was used, the time to achieve bidirectional block, though shorter, was not significantly so. However, when ECI data was visible the primary endpoint was achieved more quickly than when ECI was not visible. The use of ECI may impart this benefit by demonstrating “viable gaps” in the line when block is not achieved in the first pass. Furthermore, ECI drops during ablation, and this aids the operator in deciding how long for and when to move on to the next site. Contact force, on the other hand does not provide functional data, and it is possible that ablation may be applied with good contact but to an area that is not viable. In addition, CF technology has now been available for several years, and it may be that operators have changed the way they handle ablation catheters, paying more attention to trying to achieve contact rather than applying energy to an area which appears to have good signals. The SMART AF trial did not show any difference in time to pulmonary vein isolation between CF blinded and CF unblinded arms [13]. This may have reflected the fact that operators familiar with the use of force sensing catheters had modified their technique in the light of their experience.

The optimal ablation lesion would encompass the full thickness of myocardium with minimal damage to surrounding tissue and without the generation of a “steam pop”. This can be challenging to achieve as myocardial structure and thickness is not homogenous and titrating energy to achieve this may be difficult as it is currently not possible to easily visualise lesion formation in vivo. Measures such as ablation index [14] and lesion size index [15] have been designed to help improve the consistency of lesion formation, but provide no real-time information to the operator about the actual lesion, and an empirical value is set depending on which part of myocardium is being ablated. Ablation of non-viable myocardium is important to avoid, as it increases procedural time without enhancing efficacy, as well as risking damage to collateral structures (e.g. oesophagus). Could the combination of CF and ECI improve the quality of the ablation lesion by ensuring contact as well as ensuring that only the required amount of energy is delivered? Although this has not been tested in this trial, it is certainly an area worthy of further research. Interestingly impedance drops have been used for some time as a surrogate against which to titrate power and ensure an adequate lesion [16]. The ECI is a refinement of impedance and incorporates capacitance which is a measure of tissue viability, so that if an area of myocardium has been ablated, this will be apparent to the operator. Conversely, if an operator incorrectly believes that the catheter tip is in good contact but sees a low ECI value (due to poor contact rather than non-viable tissue), this may lead to a lack of necessary ablation. Integration of direct contact force measurement with ECI (or similar) in a single catheter would prevent this. ECI could have a role in multipolar ablation catheters such as circular ablation catheters for AF where this measurement could provide some insight into the development of the ablation lesion. ECI, however, has been shown to give high values despite demonstrably poor tissue contact in the pulmonary veins, which could conceivably lead to inappropriate ablation within the vein [4]. A reason for this may be the effect of nearby high-impedance tissue between the catheter electrode and the skin patch. A recent study in animals suggested an alternative method using a more local electrical field between catheter electrodes, which appears to counter this problem, and may be useful in future ablation technology [17].

It should be noted that despite there being no statistically significant difference in the primary endpoint between the CF non-visible group and the ECI non-visible group, the median time to achieve block in the ECI group was higher. It is unclear why this is the case, possibly operators were less familiar with the Verisense catheter (despite having experience of both technologies). This lack of familiarity may have led to slightly longer times to achieve CTI block in this group, and this may have had some effect on achieving statistical significance when the ECI information was visible. Such an effect may therefore overstate the effect of ECI information in comparison with CF information. This possible confounding should be taken into account when interpreting the results. Moreover, overall there was no difference in the achievement of bidirectional block when ECI not visible was compared to CF non visible, and ECI visible compared to CF visible.

The area of the ablation lesion in which the lowest force was applied was the tricuspid annular area. This finding was unexpected because the annular end of the lesions tends to encounter fewer problems with catheter stability compared to the venous end, where prominent anatomical features such as a Eustachian ridge can impede contact and require high degrees of catheter angulation [18]. A possible reason for poorer contact distally is that insufficient catheter reach precludes optimal contact. Selection of a different catheter curve, or the use of long venous sheaths to improve catheter support may counteract this, but such techniques were not employed frequently enough in this study to comment further.

Fluoroscopy times were shorter in the CF arm compared to ECI. This would suggest that operators may feel more confident with geometry created with the CF mapping system than that of the ECI mapping system.

A high proportion of patients (15.8%) recruited for the study were subsequently diagnosed with AF, despite the short follow–up period. AF is commonly found to co-exist or subsequently develop in AFL patients [19]. The possibility of co-existent AF should be considered in the work-up for AFL ablation as its identification is likely to alter treatment strategy. Patients must be counselled regarding the possibility of recurrent symptoms after ablation, and that these symptoms are more likely to be due to AF than recurrent AFL. When considering the anticoagulation strategy after a successful AFL ablation, the potential for AF should be taken in to account. Further research is required to identify those AFL patients most at risk of AF.

### Study limitations

Although the study met its target recruitment and pre-defined power, a larger study may have further clarified the trend to shorter ablation time in the CF cohort. The study size was limited principally by the availability of the Ensite Verisense catheters. A number of recruited patients had to be withdrawn subsequently as detailed in the results section. This was predominantly due to the presence of AF, but technical problems were also present, most notably in the ECI arm. Despite this, a significant result was obtained in this group. The presence of the high force alarm in both groups for CFs considered dangerously excessive meant that the CF not visible group was not fully blinded to CF. As switching this alarm off would have potentially compromised safety, this scientific compromise was allowed on an ethical basis.

## Conclusion

The use of tissue contact information during AFL ablation was associated with a reduction in the time taken to achieve bidirectional block when ECI was unblinded. Contact force data improved contact when visible with a trend towards a reduction in the procedural endpoint. Information on catheter tissue interaction however, does not seem to improve outcomes of flutter ablation.

## Supporting information

S1 File CONSORT 2010 Checklist.doc. (DOC)

S2 File CONSORT 2010 Flow Diagram.doc. (DOC)

S3 File IIS283 Tayebjee Protocol 2013Sep22.pdf. (PDF)

S4 File VERISMART (PLOS ONE master data final).xlsx. (XLSX)
