# Supplementary material for: Multicentre randomised trial comparing contact force with electrical coupling index in atrial flutter ablation (VERISMART trial)
Source: PLoS One. 2019 Apr 3;14(4):e0212903. doi: 10.1371/journal.pone.0212903 (PMC6447159; doi:10.1371/journal.pone.0212903)
Supplement: S2 File — (DOC) [file pone.0212903.s002.doc]

**
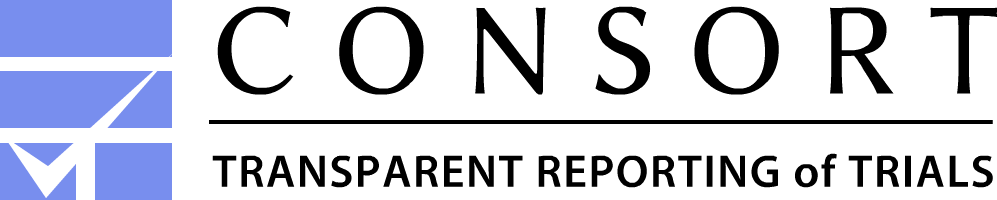
**

**CONSORT 2010 Flow Diagram**

**Allocation**

**Analysis**

**Follow-Up**

**Enrollment**

Assessed for eligibility (n=114 )

Excluded (n=16)

♦  Not meeting inclusion criteria (n=0)

♦  Declined to participate (n=0)

♦  Other reasons (n= 16)

Analysed (n= 48)
♦ Excluded from analysis (give reasons) (n= 0)

Lost to follow-up (give reasons) (n= 5)

Discontinued intervention (give reasons) (n= 0)

Allocated to intervention (n= 53)

♦ Received allocated intervention (n= 53)

♦ Did not receive allocated intervention (give reasons) (n= 0)

Lost to follow-up (give reasons) (n= 4)

Discontinued intervention (give reasons) (n=0 )

Allocated to intervention (n= 45

♦ Received allocated intervention (n= 45)

♦ Did not receive allocated intervention (give reasons) (n= 0)

Analysed (n= 40)
♦ Excluded from analysis (give reasons) (n= 1)

Randomized (n=98)
