# Supplementary material for: Multicentre randomised trial comparing contact force with electrical coupling index in atrial flutter ablation (VERISMART trial)
Source: PLoS One. 2019 Apr 3;14(4):e0212903. doi: 10.1371/journal.pone.0212903 (PMC6447159; doi:10.1371/journal.pone.0212903)
Supplement: S3 File — (PDF) [file pone.0212903.s003.pdf]

Randomised trial comparing conventional versus contact force and electrical coupling index in atrial flutter ablation (VERISMART TRIAL)

Chief Investigator : Dr MH Tayebjee , Leeds Teaching Hospitals NHS Trust

Investigators : UK Multicentre Arrhythmia Trial Group (London, Liverpool, Bristol, Plymouth, Leeds, Oxford)

BACKGROUND

Atrial Flutter

Atrial flutter (AFL) is a common cardiac rhythm abnormality, with considerable morbidity. Whilst data in the UK population is not available, US studies suggest an overall incidence of 88/100,000 person-years, rising to 587/100,000 in subjects older than 80, and being 2.5 times more common in men (FIGURE 1).[1] Extrapolation of these data to an adult UK population would suggest 35,000 new diagnoses of AFL per year. This increase in incidence with age mirrors the burden of atrial fibrillation, a closely related arrhythmic condition. AFL commonly coexists with other disease states such as hypertension, heart failure and chronic respiratory disease, adding a potentially modifiable additional burden to these conditions. It can be significantly symptomatic for the patient, negatively impacting quality of life, and the cause of considerable morbidity with persistent rapid palpitations, breathlessness, lack of energy and a need for long term rate controlling drug therapy often at the expense of side effects. Electrical cardioversion is often only a temporary respite; in one study 92% of patients had a recurrence of flutter with medical therapy or cardioversion[2].

Catheter ablation of atrial flutter

Catheter ablation for AFL is a well-established and routinely practiced treatment. Ablation is more effective than the limited options for drug therapy[3]. Acute procedural success defined by bidirectional isthmus block is more than 95%, however there is a long term recurrence rate of 5-10%[4] at a mean follow up of eight months. Bidirectional conduction block is the required endpoint for AFL ablation. The principle reason behind this is recovery of isthmus conduction[5]. Poor tissue contact and inadequate energy delivery are the main cause of this. In order to improve success rates, three dimensional mapping systems are used to allow operators to mark lesion placement, and help in the creation of a straight and complete line of ablation[4]. Until recently, catheter contact during ablation was determined using surrogates such as lack of catheter motion, electrogram attenuation and electrical impedence drops. Added information with regard to tissue contact is particularly pertinent to this procedure as anatomical variations typical to this region of the heart, with thick ridges of heart muscle and undulations of tissue, reduce the success of ablation.

Contact technology

There has been much interest in the development of technology to determine tissue catheter contact, and lesion depth. Contact information has been shown to gauge lesion size, with increased contact also being related to steam pops and thrombus formation[6]. There are currently three technologies in use. The first uses an optical fibre at the tip of the catheter to sense the contact force time integral (Tacticath, Endosense SA, Geneva, Switzerland)[7]. The second is the Thermocool Smart Touch Catheter (Biosense Webster, Diamond Bar CA, US) which has a spring at the tip of the ablation catheter which deforms as pressure is applied; this measures contact force directly. This is used in conjuction with the CARTO-3 3D mapping system (Biosense Webster, Diamond Bar CA, US) . The third technology is the Ensite Verisense System (St Jude Medical, St Paul MN, US) calculates the electrical coupling index (ECI) derived from tissue resistance and reactance[8]. This is used in conjunction with the Ensite NAVx Velocity 3D mapping system (St Jude Medical, St Paul MN, US). It is clear that the ability to quantify the catheter tissue interface is important, however there has not been a head to head comparison between contact force and ECI. Therefore it is not possible to know whether contact force or ECI have any advantages over each other or are any better than conventional techniques when considering catheter ablation of atrial flutter.

It is important to determine whether the relative advantages of each technology translate to clinical benefit. We therefore aim to investigate the differences in radiofrequency (RF) energy application time, procedure duration and outcomes between conventional ablation, contact force and ECI.

HYPOTHESES

We hypothesise that:
1. The use of either contact force or ECI will result in lower procedure time, reduced RF requirements and improved outcomes compared to the conventional approach.
2. There would be no difference in outcome measures between contact and ECI, as both provide the operator with information about the tissue catheter interface.

STUDY DESIGN

In order to examine these hypotheses we aim to perform a multicentre prospective, randomised study comparing RF energy time, procedure duration and outcome of catheter ablation of AFL between non-contact 3D guided ablation (i.e. blinded to contact or ECI sensing), contact force sensing and the assessment of ECI. Patients will be followed up for 6 months post procedure.

SCREENING PROCEDURE

A member of the site research team will screen candidates for enrolment. Subjects will be recruited from those patients listed for their first catheter ablation of AFL.

INFORMED CONSENT AND ENROLMENT

If the physician agrees, a member of the investigational site research team will discuss with the subject and any family members/significant others the study's purpose, procedures and follow-up requirements and provide written information approved by the Ethics committee prior to enrolment. All subject questions will be answered and if the subject agrees, written informed consent as approved by the Ethics Committee will be obtained. Failure to provide informed consent excludes the subject from the study. Enrolment occurs when informed consent has been signed and the patient has been randomly allocated to a treatment strategy.

INCLUSION CRITERIA

· Age ≥18 years
· Documented paroxysmal or persistent AFL

EXCLUSION CRITERIA

· Inability or unwillingness to receive oral anticoagulation
· Previous ablation procedure for AFL
· Unwillingness or inability to complete the required follow up arrangements
· Concomitant atrial fibrillation

STUDY PROTOCOL

<u>Pre –procedure</u>
Study participants will be randomized using block envelopes to undergo the procedure either blinded (control) or un-blinded to contact force or ECI. CARTO-3 or NAVx Velocity will be used as the 3D mapping systems for contact force and ECI catheters respectively. The procedures will be performed with patients in the post-absorptive state under conscious sedation or general anaesthesia.

<u>Patient preparation and procedure.</u>

As per standard practice, oral anticoagulation will be administered to the majority of patients who will then go on to have the procedure performed with uninterrupted warfarin (INR 2-3.5). Depending on randomisation, either the Ensite or CARTO-3 mapping system will be used, and the respective reference patches will be applied to the patient in the cardiac catheter laboratory. Venous sheaths will be inserted into the right and/or left femoral veins. A decapolar catheter will be inserted into the coronary sinus, and a quadripolar catheter into the right ventricle. An additional 20 pole Halo diagnostic catheter (Biosense Webster, Diamond Bar CA, US) can be placed around the tricuspid valve annulus as per operator preference. The creation of three dimensional right atrial geometry will be left to the discretion of the operator and is not a requirement.

A flutter line will be created by either a drag approach or point by point ablation using an irrigated tip Thermocool Smart Touch (Biosense Webster, Diamond Bar CA, US), or an Ensite Verisense catheter (St Jude Medical, St Paul MN, US). In the control group the operator will be blinded to contact force or ECI, by closing the respective sensing information windows on the 3D mapping system. Energy will be delivered at each point for a minimum of 30 seconds with flow limited to 17ml/min and power limited to 40W with a maximum temperature of 48°C. If bidirectional block is not achieved on the first pass, additional ablation targeting gaps will be applied. Following bidirectional block, a waiting time of 30minutes will be observed to check for, and react to, isthmus re-conduction. The procedure will end at this stage.

Data Collection

All data will be recorded on a paper case record form in the catheter laboratory, and later transferred onto an electronic database.

Post Procedure Management

Patients will be kept on telemetry for 4 hours post procedure, and mobilised following this. Patients will be discharged as per local established practice and depending on the patients well being. All anti-arrhythmic medications will be discontinued, but warfarin will be maintained for a minimum of two months.

Contact Force pressures and ECI

Because contact force sensing and ECI catheters are a new technology, there are limited data on which to base recommendations as to appropriate forces to use whilst ablating. There are some data to suggest that very high pressures are more likely to cause cardiac perforation and that the pressure required to perforate the heart is lower whilst ablating than when mapping. For the Smart Touch catheter Contact force pressures of less than 40g appear to be safe with a margin of error that is likely to be large (i.e. pressures considerably higher than this are probably also safe in the majority of cases). Conversely, low pressures are more likely to be associated with ineffective lesion formation. In order to try to ensure safe and effective use of the Smart Touch catheter, a contact force range of 5–40g will be recommended. For the Verisense catheter a change in ECI of 12% will be aimed for, as this appears to be the optimal value for safe full thickness lesion formation[8]. Values above 12% have been associated with collateral tissue injury. A visual warning on the CARTO navigation system will be programmed to occur if pressures exceed 50g. Early studies have shown wide intra-operator variability in terms of level of force used, and pressures can be affected by factors such as cardiac contraction and where the catheter is positioned anatomically. Therefore the final decision about whether to ablate at any given contact force measurement and power setting will be left to the discretion of the operator. As further evidence emerges as to the ideal pressures and ECI index to use, it is likely that a range of pressures will be recommended but this range will not be prescriptive for the study.

Follow up

Patients will be followed up at three and six months post procedure. A seven day ambulatory ECG will be recorded prior to the follow up visit. All patients who experience a recurrence will be offered a repeat electrophysiological study if clinically indicated.

Outcome measures

Primary outcome: Time to achieve bi-directional block(secs) . This is defined as the time from the first lesion to the time that consistent (> one minute) bidirectional block is achieved.

Total RF energy required for the whole procedure (sec), and total energy required for ablation (power x ablation time in secs).

Secondary outcomes: complications during initial hospitalisation and during six month follow up, isthmus re-conduction during the 30 minute waiting period, total procedure time, procedural cost, fluoroscopy time,

radiation exposure, total radiofrequency energy delivered, and long term success rate (freedom from AFL episodes of >30seconds duration following discontinuation of anti-arrhythmic medication), isthmus re-conduction at repeat procedure.

Sample Size

Previous studies have focussed on number of RF lesions (mean typically 12 – 19) and total procedural time (mean typically 100-150mins), along with fluoroscopy time (mean typically 2-10 mins)[9]. No local data is available to guide timings for bidirectional block using different catheter technologies and mapping systems. Therefore based on prior publications, a sample size of 120 patients is sufficient for this feasibility study i.e. 30 patients in each group[10].

Statistical methods

Continuous variables will be checked for normality. If they are normally distributed the results will expressed as mean ± SD and compared by Student's t-test or ANOVA. If they are skewed the results will be expressed as median and interquartile range, and compared using the Mann Whitney U test. A chi-squared test will be used to compare categorical variables. A p value <0.05 will be considered statistically significant.
Alternative statistical methods and additional analyses will be conducted as deemed appropriate.

RISKS AND EXPECTED VALUE OF RESULTS

Potential Risks

All subjects included in the study will be exposed to the risks of catheter ablation. These include minor risks (spontaneous resolution without treatment) of femoral bruising, chest pain and palpitation. Major risks (require treatment) include procedure failure resulting in recurrent sustained atrial arrhythmia (<5%), pericardial effusion leading to tamponade (<1%), stroke (<1%), femoral vascular complications (1%), and AV node damage requiring pacing (<1%). As subjects will have already been assessed by an expert as clinically indicated to have catheter ablation for AFL these risks are not directly related to the study. All subjects will routinely be informed of these risks and provide written informed consent for the procedure as per standard practice. The techniques and treatment strategies used for the study are identical to standard clinical ablation procedures.

Expected value of results

There is evidence that catheter ablation for AFL is superior to medical therapy both in terms of clinical and economic outcomes[11], and AFL ablation is now considered as routine practice. Further, what is becoming evident is that knowledge of tissue catheter interaction reduces procedure time and can improve outcomes. What is unclear is whether the individual differences between contact technologies offer specific clinical advantages, and this trial will provide insight into this. In addition, information about the catheter tissue interface may make the procedures safer as high forces that can potentially traumatise myocardium can be avoided. Finally, the ability to tell that the catheter is delivering effective energy to tissue can potentially shorten procedure times allowing more patients to be treated. The results are potentially applicable to catheter ablation of atrial fibrillation and ventricular tachycardia.

FUNDING

Each catheter will add £300 per procedure. In addition for administration (e.g. research nurse time) we anticipate a further cost of £100 per patient. Therefore the total funding sought is £48,000.

Figure 1. Incidence of Atrial Flutter with age[1]

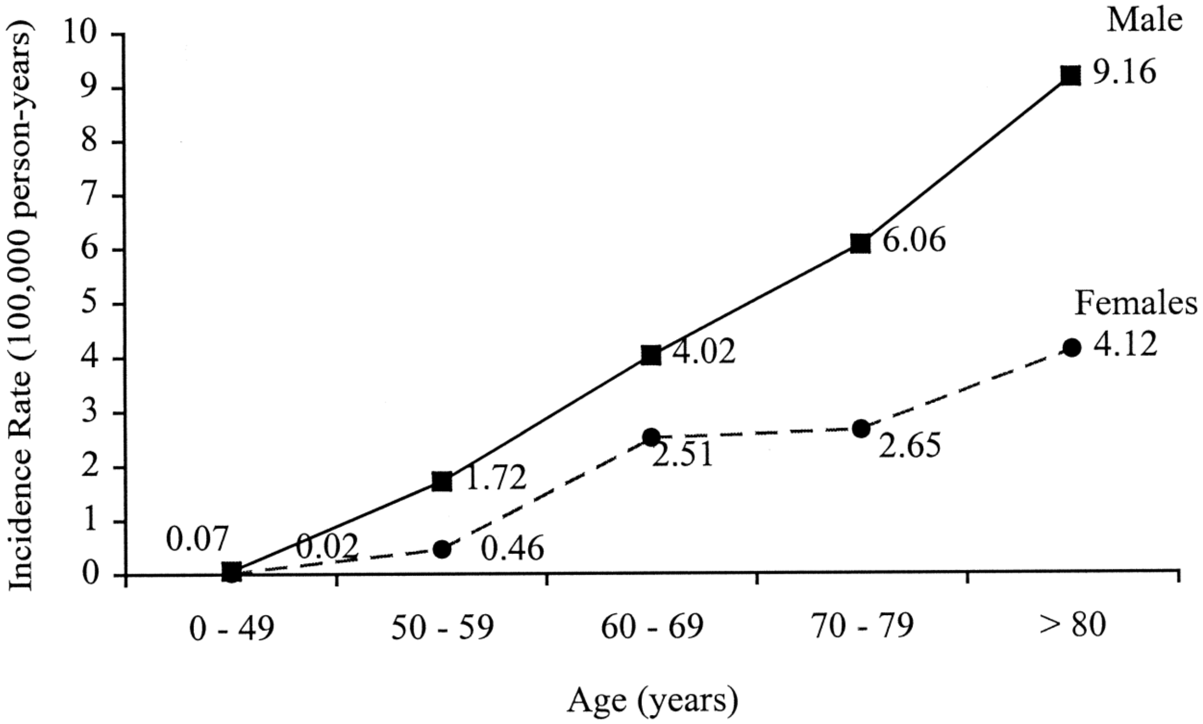

Figure 2. Randomisation process

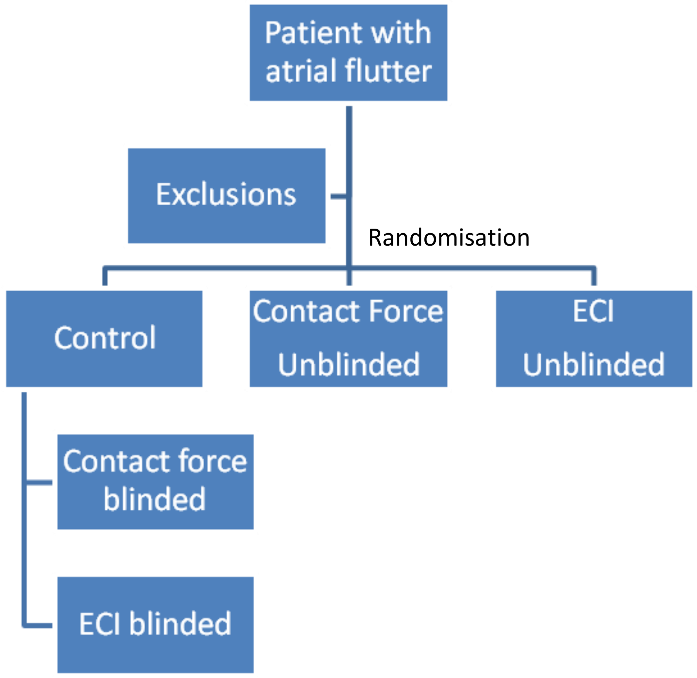

REFERENCES

[1] Juan Granada, William Uribe, Po-Huang Chyou, Karen Maassen, Robert Vierkant, Peter N Smith, John Hayes, Elaine Eaker, Humberto Vidaillet, Incidence and predictors of atrial flutter in the general population, Journal of the American College of Cardiology, Volume 36, Issue 7, December 2000, Pages 2242-2246

[2] Babaev A, Suma V, Tita C, Steinberg J. Recurrence rate of atrial flutter after initial presentation in patients on drug treatment. Am J Cardiol. 2003;92:1122-4.

[3] A Natale, KH Newby, E Pisano et al. Prospective randomized comparison of antiarrhythmic therapy versus first-line radiofrequency ablation in patients with atrial flutter. J Am Coll Cardiol 2000;35: 1898–1904.

[4] Willems S, Weiss C, Ventura R, Rüppel R, Risius T, Hoffmann M, Meinertz T. Catheter ablation of atrial flutter guided by electroanatomic mapping (CARTO): a randomized comparison to the conventional approach. J Cardiovasc Electrophysiol. 2000;11:1223-30.

[5] Lehrmann H, Weber R, Park CI, Allgeier J, Schiebeling-Römer J, Arentz T, Jadidi A. "Dormant transisthmus conduction" revealed by adenosine after cavotricuspid isthmus ablation. Heart Rhythm. 2012;9:1942-6.

[6]K. Yokoyama, H. Nakagawa, D.C. Shah et al. Novel CF sensor incorporated in irrigated radiofrequency ablation catheter predicts lesion size and incidence of steam pop and thrombus. Circ Arrhythmia Electrophysiol. 2008; 1:354–362.

[7] D.C. Shah, H. Lambert, H. Nakagawa, A. Langenkamp, N. Aeby, G. Leo. Area under the real-time contact force curve (force-time integral) predicts radiofrequency lesion size in an in vitro contractile model simulating beating heart. J Cardiovasc Electrophysiol. 2010;21: 1038–1043.

[8] Homes D, Fish J, Byrd I, Dando J, Fowler S, Cao H, Jensen J, Puryear H, Chinitz H. Contact Sensing Provides a Highly Accurate Means to Titrate Radiofrequency Ablation Depth. J Cardiovasc Electrophysiol. 2011;22: 684-90.

[9] Ventura R, Rostock T, Willems S, et al. Catheter ablation of common-type atrial flutter guided by three-dimensional right atrial geometry reconstruction and catheter tracking using cutaneous patches: a randomized prospective study. *Journal Of Cardiovascular Electrophysiology*. October 2004;15(10):1157-1161.

[10] Lancaster GA, Dodd S, Williamson P. Design and analysis of pilot studies: recommendations for good practice. Journal of Evaluation in Clinical Practice,10:307–312

[11] Rodgers M, McKenna C, Palmer S, Chambers D, Van Hout S, Golder S, Pepper C, Todd D, Woolacott N. Curative catheter ablation in atrial fibrillation and typical atrial flutter: systematic review and economic evaluation. Health Technol Assess. 2008 Nov;12(34): iii-iv, xi-xiii, 1-198
